# The prognostic role of platelet-to-lymphocyte ratio on overall survival in gastric cancer: a systematic review and meta-analysis

**DOI:** 10.1186/s12876-020-1167-x

**Published:** 2020-01-20

**Authors:** Weijuan Cao, Xiaomin Yao, Danwei Cen, Yajun Zhi, Ningwei Zhu, Liyong Xu

**Affiliations:** 1 College of Pharmacy, Zhejiang Pharmaceutical College, Ningbo, 315100 Zhejiang Province China; 2 Zhejiang Pharmaceutical College, Ningbo Higher Education Park, No 888, East Section of Yinxian Avenue, Ningbo, 315100 Zhejiang Province China

**Keywords:** Prognosis, Blood platelets, Lymphocyte count, Meta-analysis, Stomach neoplasms

## Abstract

**Background:**

This study aimed to summarize the previously published literature on the role of platelet-to-lymphocyte ratio (PLR) on overall survival (OS) in patients with gastric cancer.

**Methods:**

We systematically searched PubMed, EmBase, and the Cochrane library to identify eligible studies to review. Pooled hazard ratios (HRs) and 95% confidence intervals (CIs) were calculated using the random-effects model. Sensitivity and subgroup analyses were performed, and publication bias was assessed.

**Results:**

A total of 28 studies comprising 15,617 patients with gastric cancer were included in this meta-analysis. The pooled results indicated that elevated PLR was associated with poor OS (HR: 1.37; 95% CI: 1.24–1.51; *P* < 0.001). A significant publication bias was observed (Egger test, *P* = 0.036; Begg test, *P* = 0.017). After adjusting for publication bias using the trim and fill method, an adjusted pooled HR of 1.19 (95% CI: 1.08–1.33; *P* = 0.001) was observed. Subgroup analyses indicated an elevated PLR in retrospective studies. Studies conducted in Turkey, the UK, the USA, and Costa Rica; studies with a sample size of < 1000, with < 70% male patients, and with patients treated with chemotherapy; studies with PLR cutoff value of ≥200; and studies with lower quality as determined by the Newcastle-Ottawa Scale all showed greater harmful effects on OS than their corresponding subsets (*P* < 0.05).

**Conclusions:**

An elevated PLR was associated with poor OS in patients with gastric cancer. These results might differ between studies due to differences in design, country of origin, sample size, sex proportion, treatment strategy, PLR cutoff value, and study quality.

## Background

Gastric cancer is the second most common cancer in China. Nearly 679,100 new gastric cancer cases are diagnosed, and 498,000 patients die from gastric cancer annually [1]. Patients are usually diagnosed at advanced or metastatic stages due to the lack of clinical symptoms specific to gastric cancer, making it an extremely deadly disease with unfavorable prognosis despite the development of new surgical techniques, chemotherapy, and radiotherapy [2]. Currently, the standard treatment strategy for metastatic gastric cancer includes chemotherapy and targeted therapy. The response rate to first-line treatment ranges from 27 to 54% [3–5]. Therefore, simple, low-cost methods to evaluate the prognosis of gastric cancer should be explored.

Several studies have indicated that the immune system can affect tumor growth, with neutrophils, lymphocytes, monocytes, and platelets possibly playing an important role in the tumor-induced systemic inflammatory response [6, 7]. This response may accelerate tumor development and metastasis through the following mechanisms: promoting secretion of inflammatory mediators and cytokines, inhibiting apoptosis, and damaging the tumor cell DNA [8]. Previous meta-analyses have already demonstrated the prognostic role of platelet-to-lymphocyte ratio (PLR) in gastrointestinal cancers [9–11], and whether this association differs according to patients’ characteristics remains controversial. Therefore, this study was conducted to update the magnitude for the role of PLR on overall survival (OS) of patients with gastric cancer. Moreover, differences in this association based on patients’ characteristics were also investigated.

## Methods

### Data sources, search strategy, and selection criteria

This review was conducted and reported according to the Preferred Reporting Items for Systematic Reviews and Meta-Analysis Statement issued in 2009 [12]. Studies that investigated the role of PLR on OS in patients with gastric cancer were eligible for inclusion in this meta-analysis, with no restriction on language of publication. Utilizing the Boolean logic, the core search template in PubMed, EmBase, and the Cochrane library was [(“PLR” OR “platelet lymphocyte ratio”) AND (“gastric cancer” OR “stomach cancer”) AND (“prognosis” OR “survival”)]. Each database was searched from its date of inception through November 2018. Manual searches of the reference lists of eligible studies and relevant reviews obtained in the database search were also carried out to identify any relevant new studies or studies otherwise missed.

Two independent reviewers conducted the literature search and study selection process following a standardized flowchart. The inclusion criteria for this meta-analysis were as follows: 1) patients: all studies including patients diagnosed with gastric cancer, irrespective of stage; 2) comparison: all studies comparing high PLR with low PLR; and 3) outcome: all studies reporting OS. Furthermore, studies designed as either prospective or retrospective were included, whereas those including patients with secondary cancers in addition to gastric cancer were excluded.

### Data collection and quality assessment

Data collection and quality assessment were performed by two independent reviewers, and any inconsistencies or disputes were settled by a third independent reviewer. Collected data from each study included the first author’s name, year of publication, study design, country of origin, sample size, sex proportion and mean age of the study cohort, treatment strategy, disease status, cutoff value of PLR used to define elevated level, and OS. The quality of each study was evaluated using the Newcastle-Ottawa Scale (NOS) which consists of the following 3 subscales: selection (4 items), comparability (1 item), and outcome (3 items). The NOS is quite comprehensive and has been partially validated for evaluating the quality of observational studies in meta-analysis [13]. The “star system” of NOS ranges from 0 to 9; studies with 7–9 stars are considered high quality, whereas those with ≤6 stars are considered low quality.

### Statistical analysis

The prognostic role of PLR on OS for patients with gastric cancer was analyzed by abstracting the hazard ratios (HRs) and 95% confidence intervals (CIs) reported in each individual study. The pooled results were then calculated using the random-effects model, which considers that the true underlying effect varies across included studies [14, 15]. Heterogeneity among included studies was calculated using the I-square and Q statistics, with I-square of > 50.0% or *P* < 0.10 indicating significant heterogeneity [16, 17]. Then, sensitivity analysis then performed to assess the stability of pooled results [18]. Subgroup analyses were also conducted to evaluate the relationship between PLR and OS according to the study design, country of origin, sample size, sex proportion and mean age of cohort, treatment strategy, disease status, PLR cutoff value, and NOS score. *P*-values between subgroups were also calculated using the interaction t-test [19]. Publication bias was investigated with qualitative and quantitative methods, including funnel plot, Egger test [20], and Begg test [21]. *P*-values for pooled results were two-sided, and the inspection level was 0.05. All statistical analyses were computed with STATA software (version 10.0; Stata Corporation, College Station, TX, USA).

## Results

### Literature search

**Fig. 1:**
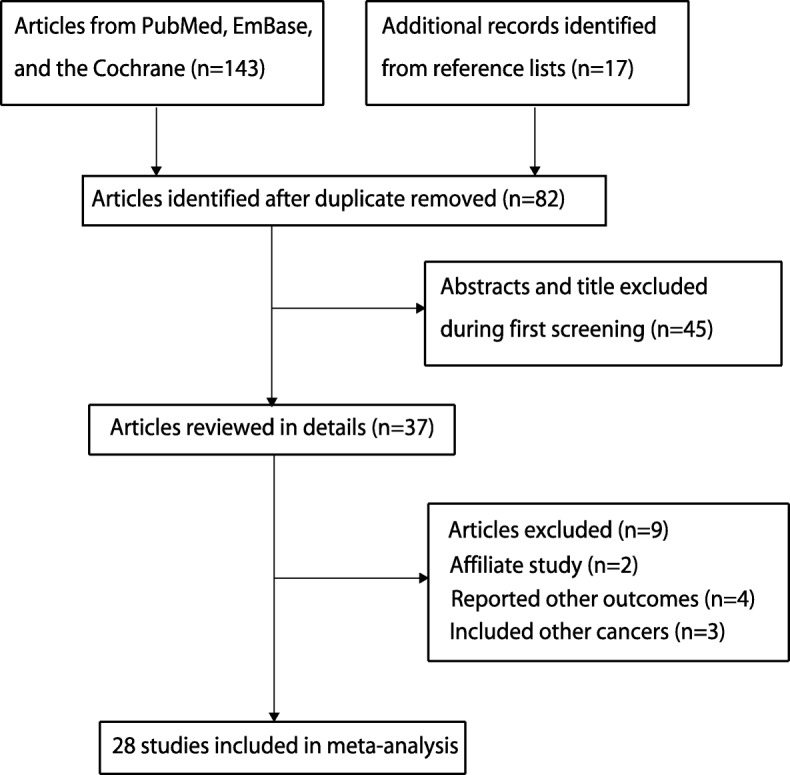
A flow diagram of the literature search and trials selection process

The initial search in the 3 electronic databases identified 143 studies, of which 106 were excluded due to duplication and irrelevance to this meta-analysis. Thirty-seven potentially eligible studies were selected for further evaluation; 9 were excluded due to the following reasons: same study population (*n* = 2), OS not reported as outcome (*n* = 4), and secondary cancers were included (*n* = 3). Manual searches of the reference lists of these studies identified 17 articles, and all of them were already included in the initial electronic searches. Finally, 28 studies were selected for meta-analysis [22–49]. The study selection process is presented as PRISMA flowchart in Fig. 1, and the baseline characteristics of the included studies are shown in Table 1.

**Table 1:** Baseline characteristics of the selected studies

Study	Publication year	Study design	Country	Sample size	Percent of male (%)	Mean age (years)	Treatment strategy	Disease status	Cutoff value of PLR	NOS score
Aliustaoglu [23]	2010	Retrospective	Turkey	168	67.8	60.1	Chemotherapy	Advanced	160	6
Lee [32]	2013	Retrospective	Korea	174	65.5	55.0	Chemotherapy	Advanced	160	8
Jiang [30]	2014	Prospective	China	377	67.1	64.0	Surgery	Early	184	7
Wang [45]	2014	Retrospective	China	439	72.7	NA	Mixed	Advanced	160	7
Lian [33]	2015	Retrospective	China	162	69.8	56.3	Surgery	All	208	8
Aldemir [22]	2015	Retrospective	Turkey	103	56.3	58.0	Mixed	Early and Advanced	170	7
Deng [24]	2015	Retrospective	China	389	72.5	65.0	Surgery	All	132	8
Gunaldi [28]	2015	Retrospective	Turkey	245	72.2	59.6	Mixed	All	160	7
Hsu [29]	2015	Retrospective	China	1030	64.5	NA	Surgery	All	132	7
Kim [31]	2015	Prospective	Korea	1986	66.3	58.2	Surgery	Early	126	7
Liu [34]	2015	Retrospective	China	455	69.0	59.0	Surgery	Early	188	6
Sun [39]	2015	Retrospective	China	632	65.3	57.0	Surgery	All	140	7
Wang [42]	2015	Retrospective	China	120	62.5	68.0	Chemotherapy	Advanced	235	8
Feng [25]	2016	Retrospective	China	3243	78.3	57.3	Mixed	Advanced	130	7
Sun [40]	2016	Retrospective	China	305	66.2	57.0	Surgery	Early	120	8
Zhou [49]	2016	Retrospective	China	451	71.8	NA	Surgery	Early	255	7
Wen [46]	2017	Retrospective	UK	253	66.1	75.5	Surgery	All	150	6
Fuentes [26]	2017	Retrospective	USA	112	66.1	58.0	Mixed	Advanced	260	6
Song [38]	2017	Retrospective	China	1990	73.7	62.0	Surgery	Advanced	139	7
Wang [43]	2017	Retrospective	China	273	68.1	56.7	Chemotherapy	Advanced	202	6
Wang [44]	2017	Retrospective	China	444	63.3	56.0	Surgery	All	120	7
Ramos-Esquivel [36]	2018	Retrospective	Costa Rica	381	57.2	61.2	Mixed	All	350	7
Petrillo [35]	2018	Retrospective	Italy	151	64.2	62.0	Chemotherapy	Advanced	157	8
Saito [37]	2018	Retrospective	Japan	453	73.1	67.7	Surgery	All	173	7
Gong [27]	2018	Retrospective	China	91	75.8	55.0	Mixed	Advanced	108	7
Zhang [48]	2018	Retrospective	China	182	67.0	65.0	Mixed	All	172	7
Tang [41]	2018	Retrospective	China	104	71.2	NA	Chemotherapy	Advanced	131	6
Zhang [47]	2018	Retrospective	China	904	74.4	NA	Surgery	All	160	7

### Study characteristics

Two prospective and 26 retrospective studies reporting a total of 15,617 patients with gastric cancer were included in this meta-analysis. The sample size ranged from 91 to 3243, and the proportion of male patients ranged from 56.3 to 78.3%. Eighteen studies were conducted in China, 3 in Turkey, 2 in Korea, 1 in Japan, 1 in the UK, 1 in the USA, 1 in Costa Rica, and 1 in Italy. Fourteen studies included patients treated with surgery, 6 with chemotherapy, and the remaining 8 included patients who received combined treatment strategies. Five studies included patients in early stages, 11 with advanced stages, and the remaining 12 with all stages. The mean patient age in the included studies ranged from 55.0 to 75.5 years, and the PLR cutoff value used to define elevated level ranged from 108 to 305. Study quality was evaluated using the NOS: 6 studies had 8 stars, 16 had 7 stars, and the remaining 6 had 6 stars.

### Meta-analysis and sensitivity analysis

**Fig. 2:**
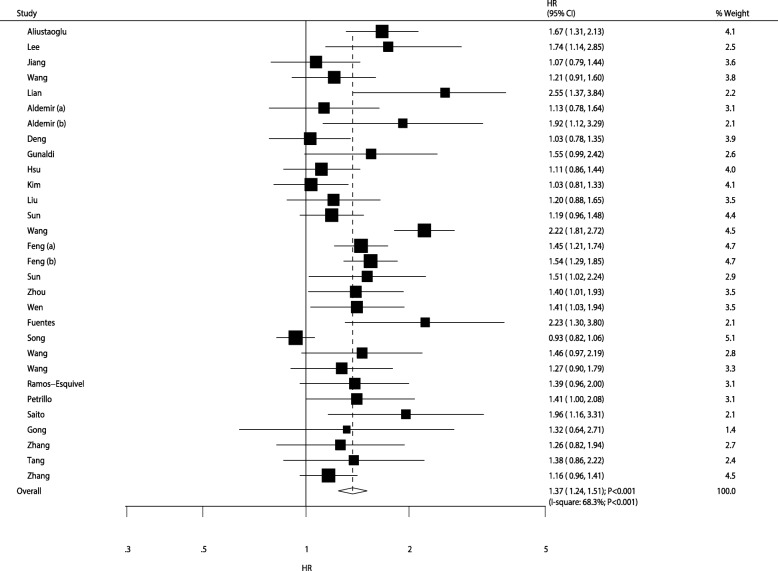
The prognostic role of PLR on OS in gastric cancer patients

After pooling all included studies, patients with gastric cancer with an elevated PLR were noted to have lower OS than those with lower PLR level (HR: 1.37; 95% CI: 1.24–1.51; *P* < 0.001; Fig. 2). Significant heterogeneity among the included studies was observed (I-square: 68.3%; P < 0.001). The results of sensitivity analyses are presented in Table 2; we noted that higher PLR was associated with lower OS in the pooled conclusion. Moreover, studies conducted by Wang et al. [42] and Song et al. [38] were noted to be responsible for most of the heterogeneity in the summary results.

**Table 2:** Sensitivity analysis for overall survival

Excluding study	Including studies	HR and 95% CI	*P* value	Heterogeneity (%)	P value for heterogeneity
Aliustaoglu [23]	22,24–49	1.36 (1.23–1.50)	< 0.001	67.9	< 0.001
Lee [32]	22–31,33–49	1.36 (1.23–1.50)	< 0.001	68.8	< 0.001
Jiang [30]	22–29,31–49	1.38 (1.25–1.53)	< 0.001	68.8	< 0.001
Wang [45]	22–44,46–49	1.38 (1.25–1.52)	< 0.001	69.3	< 0.001
Lian [33]	22–32,34–49	1.35 (1.23–1.49)	< 0.001	67.0	< 0.001
Aldemir [22] (a)	23–49	1.38 (1.25–1.52)	< 0.001	69.2	< 0.001
Aldemir [22] (b)	23–49	1.36 (1.23–1.50)	< 0.001	68.7	< 0.001
Deng [24]	22,23,25–49	1.39 (1.25–1.53)	< 0.001	68.3	< 0.001
Gunaldi [28]	22–27,29–49	1.37 (1.24–1.51)	< 0.001	69.2	< 0.001
Hsu [29]	22–28,30–49	1.38 (1.25–1.53)	< 0.001	68.8	< 0.001
Kim [31]	22–30,32–49	1.39 (1.25–1.53)	< 0.001	68.2	< 0.001
Liu [34]	22–33,35–49	1.38 (1.25–1.52)	< 0.001	69.3	< 0.001
Sun [39]	22–38,40–49	1.38 (1.25–1.53)	< 0.001	69.1	< 0.001
Wang [42]	22–41,43–49	1.33 (1.22–1.45)	< 0.001	55.8	< 0.001
Feng [25] (a)	22–24,26–49	1.37 (1.23–1.52)	< 0.001	68.9	< 0.001
Feng [25] (b)	22–24,26–49	1.36 (1.23–1.51)	< 0.001	68.1	< 0.001
Sun [40]	22–39,41–49	1.37 (1.24–1.51)	< 0.001	69.2	< 0.001
Zhou [49]	22–48	1.37 (1.24–1.52)	< 0.001	69.3	< 0.001
Wen [46]	22–45,47–49	1.37 (1.24–1.51)	< 0.001	69.3	< 0.001
Fuentes [26]	22–25,27–49	1.36 (1.23–1.49)	< 0.001	68.0	< 0.001
Song [38]	22–37,39–49	1.39 (1.28–1.52)	< 0.001	54.1	< 0.001
Wang [43]	22–42,44–49	1.37 (1.24–1.51)	< 0.001	69.3	< 0.001
Wang [44]	22–43,45–49	1.37 (1.24–1.52)	< 0.001	69.3	< 0.001
Ramos-Esquivel [36]	22–35,37–49	1.37 (1.24–1.51)	< 0.001	69.3	< 0.001
Petrillo [35]	22–34,36–49	1.37 (1.24–1.51)	< 0.001	69.3	< 0.001
Saito [37]	22–36,38–49	1.36 (1.23–1.50)	< 0.001	68.5	< 0.001
Gong [27]	22–26,28–49	1.37 (1.24–1.51)	< 0.001	69.4	< 0.001
Zhang [48]	22–47,49	1.37 (1.24–1.52)	< 0.001	69.3	< 0.001
Tang [41]	22–40,42–49	1.37 (1.24–1.51)	< 0.001	69.3	< 0.001
Zhang [47]	22–46,48,49	1.38 (1.25–1.53)	< 0.001	68.9	< 0.001

### Subgroup analysis

Subgroup analyses for the prognostic role of PLR on OS in gastric cancer are presented in Table 3 and Additional file 1: Figures S1, S2, S3, S4, S5, S6, S7, S8 and S9. Increased PLR was found to be associated with lower OS in gastric cancer in most subsets. However, PLR was not significantly associated with OS in prospectively designed studies, nor in studies conducted in Japan and Korea. When comparing relative ratios between subgroups, PLR was found to be higher in the pooled results from retrospective studies. Studies conducted in Turkey, the UK, the USA, and Costa Rica; studies with sample size of < 1000; studies including < 70% male patients; studies with patients treated with chemotherapy; studies with PLR cutoff value ≥200; and studies of lower quality as determined by the NOS score all showed greater harmful effects on OS as compared to their corresponding subgroups (Table 2).

**Table 3:** Subgroup analysis for overall survival

Factor	Groups	Number of cohorts	HR and 95% CI	*P* value	Heterogeneity (%)	*P* value for heterogeneity	*P* value between subgroups
Study design	Prospective	2	1.05 (0.87–1.27)	0.625	0.0	0.868	0.022
	Retrospective	28	1.40 (1.26–1.55)	< 0.001	68.6	< 0.001	
Country	China	19	1.32 (1.16–1.49)	< 0.001	75.0	< 0.001	0.045
	Japan or Korea	3	1.45 (0.94–2.25)	0.092	71.4	0.030	
	Other	8	1.51 (1.33–1.72)	< 0.001	0.0	0.503	
Sample size	≥ 1000	11	1.25 (1.09–1.45)	0.002	69.2	< 0.001	< 0.001
	< 1000	19	1.44 (1.28–1.63)	< 0.001	60.4	< 0.001	
Percent male	≥ 70.0	10	1.31 (1.10–1.55)	0.002	72.9	< 0.001	0.014
	< 70.0	20	1.40 (1.25–1.58)	< 0.001	63.5	< 0.001	
Mean age (years)	≥ 60.0	13	1.41 (1.16–1.71)	0.001	82.2	< 0.001	0.168
	< 60.0	12	1.39 (1.23–1.57)	< 0.001	41.6	0.064	
Treatment strategy	Surgery	14	1.21 (1.08–1.35)	0.001	56.5	0.005	< 0.001
	Chemotherapy	6	1.70 (1.43–2.03)	< 0.001	40.4	0.136	
	Mixed	10	1.44 (1.31–1.59)	< 0.001	0.0	0.545	
Disease status	Early	6	1.18 (1.04–1.34)	0.012	0.0	0.533	0.076
	Advanced	13	1.51 (1.26–1.82)	< 0.001	82.0	< 0.001	
	All	11	1.29 (1.14–1.45)	< 0.001	35.1	0.118	
Cutoff value	≥ 200	6	1.79 (1.43–2.24)	< 0.001	56.8	0.041	< 0.001
	< 200	24	1.28 (1.17–1.40)	< 0.001	53.9	0.001	
NOS scale	High	24	1.34 (1.20–1.50)	< 0.001	72.0	< 0.001	0.039
	Low	6	1.49 (1.30–1.72)	< 0.001	0.0	0.416	

### Publication Bias

**Fig. 3:**
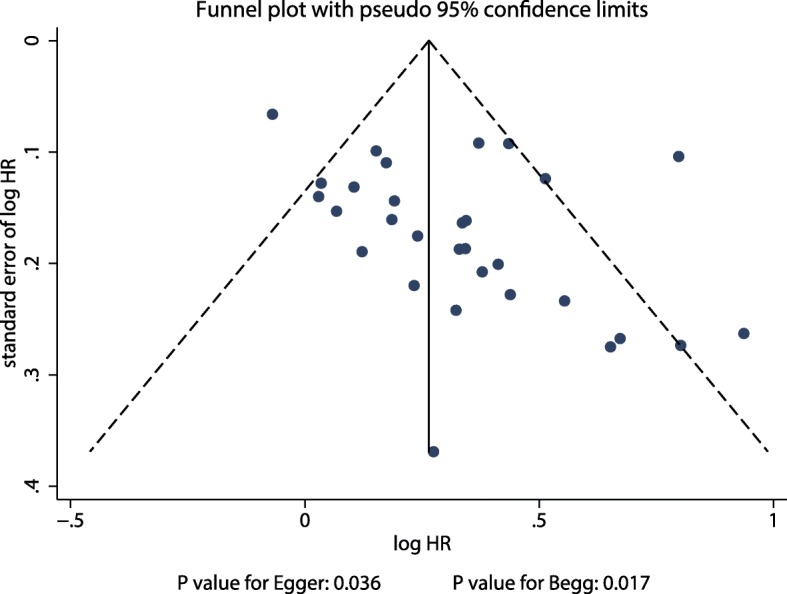
Publication bias for the prognostic role of PLR on OS in gastric cancer patients

**Fig. 4:**
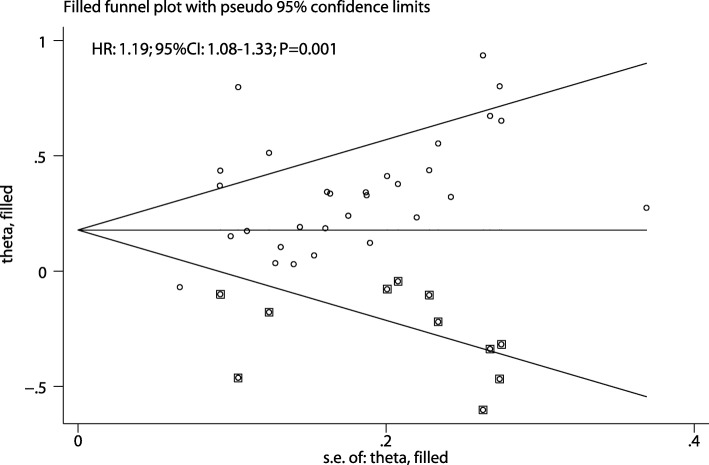
The pooled result adjusted by the trim and fill method

Publication bias for the prognostic role of PLR on OS in gastric cancer was assessed and is presented in Fig. 3. Results of the Egger and Begg tests showed significant publication bias (*P* = 0.036 and *P* = 0.017, respectively). Our finding that elevated PLR is associated with lower OS did not change after the adjustment for publication bias using the trim and fill method [50]. The adjusted pooled HR was 1.19 (95% CI: 1.08–1.33; *P* = 0.001; Fig. 4).

## Discussion

The current meta-analysis was based on all published observational studies that explored the prognostic role of PLR on OS in gastric cancer, and the prognostic ability of elevated PLR on OS was compared between subgroups based on pre-defined factors. This comprehensive quantitative meta-analysis comprised a total of 15,617 patients with gastric cancer from 2 prospective and 26 retrospective studies with a wide range of study and patient characteristics. The pooled results indicated that elevated PLR was significantly associated with lower OS in gastric cancer. This result is stable and not altered by excluding any specific study from the analysis. The results of subgroup analyses indicated that elevated PLR predicted poor OS in most subsets. In the pooled retrospective studies, elevated PLR was noted to cause greater harmful effects on OS than their corresponding subgroups in studies conducted in Turkey, the UK, the USA, and Costa Rica; studies with sample size of < 1000; studies with < 70% male patients; studies with patients treated with chemotherapy; studies with PLR cutoff value of ≥200; and studies of lesser quality.

A previous meta-analysis based on 13 studies found that elevated PLR was associated with poor OS, but without significant effect on disease-free survival [51]. Subgroup analyses indicated that the prognostic roles of PLR on OS differed based on race, treatment strategy, disease status, and cutoff value of PLR. However, data from these included studies were assessed and revealed that some data were not consistent with that of the original study. The study conducted by Zhou et al. indicated an elevated PLR was not significantly associated with OS in patients with gastric cancer according to 3 studies [52]. Moreover, Xu et al. conducted a meta-analysis of 8 studies and concluded an elevated PLR was not associated with OS in patients with gastric cancer, but was significantly correlated with greater risk of lymph node metastasis, serosal invasion, and advanced stage risk [53]. However, stratified analyses according to some characteristics, including the mean age of patients, sex proportion, and study quality, were not addressed. Furthermore, numerous relevant studies were published in 2017 and 2018, but were not yet included in any pooled results. Therefore, this meta-analysis was conducted to thoroughly evaluate the prognostic role of PLR on OS in gastric cancer and include newer updated studies.

The pooled results indicated that elevated PLR was significantly associated with poor OS in gastric cancer. However, several studies included in the meta-analysis did not observe this. Jiang et al. showed that neutrophil–lymphocyte ratio (NLR) and PLR are prognostic factors for operable gastric cancer, whereas PLR was not a prognostic factor for OS [30]. Wang et al. found the median survival time in patients with PLR of > 160 and PLR of < 160 as 8.5 months and 10 months, respectively; this small difference was not statistically significant [45]. Aldemir et al. found that PLR could not significantly predict OS in patients with early-stage gastric cancer but could in those with advanced gastric cancer [22]. Deng et al. suggested that preoperative PLR was significantly correlated with tumor progression and poor prognosis in patients with gastric cancer after a surgical resection [24]. Gunaldi et al. found no significant association between PLR and OS in gastric cancer of all stages [28]. Hsu et al. used PLR of 132 as the cutoff value and found that elevated PLR was not associated with OS in gastric cancer at all stages [29]. Similarly, the study conducted by Kim et al. suggested that PLR and NLR were associated with gastric cancer prognosis and indicated that NLR was more predictive of OS than PLR [31]. Several other studies also did not find elevated PLR to be associated with OS in patients with gastric cancer [27, 34, 36, 38, 39, 41, 43, 44, 47, 48]. These results might vary due to the study design, disease stage, and cutoff values of PLR. Differences between studies in median survival rates might be biased due to the relationship of PLR with tumor size and disease stage.

Subgroup analyses indicated that the prognostic role of PLR on OS in gastric cancer might be affected by the study design, country of origin, sample size, sex proportion, treatment strategy, cutoff values of PLR, and study quality. This condition potentially occurs due to the following reasons: 1) the number of included studies was not balanced between subgroups, which might affect the pooled results; 2) weighted pooled results could affect the prognostic role of PLR on OS in patients with specific characteristics; 3) background therapies and tumor stage are associated with the prognosis of patients with gastric cancer; and 4) study quality was correlated with evidence level and reliability of pooled results.

Although this study provided a comprehensive meta-analysis for the prognostic role of PLR on OS in gastric cancer, several limitations should be acknowledged: 1) most studies included were retrospective in design, which might introduce confounding variables, thus overestimating the pooled result; 2) different adjusted models, treatment strategies, and tumor stages among included patients might introduce a substantial heterogeneity among the included studies; 3) a significant publication bias among the included studies was observed, although the adjusted result was calculated; and 4) individual data were not available and more detailed analyses not conducted.

## Conclusion

In conclusion, the pooled result indicated that elevated PLR was associated with poor OS in patients with gastric cancer. Moreover, the adjusted HR indicated decreased harmful effects after adjusting for potential publication bias. Furthermore, the prognostic role of PLR on OS might be affected by the study design, country of origin, sample size, treatment strategy, cutoff values of PLR, and study quality. Further large-scale prospective studies should be conducted to verify the findings in this study and evaluate the role of PLR on the prognosis (progression-free survival and disease-free survival) of gastric cancer.

## Supplementary information

**Additional file 1.** Subgroup analyses for overall survival. Subgroup analyses for overall survival based on study design, country, sample size, percent male, mean age, treatment strategy, disease status, cutoff value and study quality.

## Data Availability

All data generated or analyzed during this study are included in this published article and its supplementary information files.

## Abbreviations

CIs: Confidence intervals
HRs: Hazard ratios
NLR: Neutrophil–lymphocyte ratio
NOS: Newcastle-Ottawa Scale
OS: Overall survival
PLR: Platelet-to-lymphocyte ratio

## References

[1] Chen W, Zheng R, Baade PD, Zhang S, Zeng H, Bray F, Jemal A, Yu XQ, He J (2016). Cancer statistics in China, 2015. CA Cancer J Clin.

[2] Ma J, Yao S, Li XS, Kang HR, Yao FF, Du N (2015). Neoadjuvant therapy of DOF regimen plus Bevacizumab can increase surgical resection Ratein locally advanced gastric Cancer: a randomized, controlled study. Medicine (Baltimore).

[3] Bang YJ, Van Cutsem E, Feyereislova A, Chung HC, Shen L, Sawaki A, Lordick F, Ohtsu A, Omuro Y, Satoh T (2010). Trastuzumab in combination with chemotherapy versus chemotherapy alone for treatment of HER2-positive advanced gastric or gastro-oesophageal junction cancer (ToGA): a phase 3, open-label, randomised controlled trial. Lancet.

[4] Koizumi W, Kim YH, Fujii M, Kim HK, Imamura H, Lee KH, Hara T, Chung HC, Satoh T, Cho JY (2014). Addition of docetaxel to S-1 without platinum prolongs survival of patients with advanced gastric cancer: a randomized study (START). J Cancer Res Clin Oncol.

[5] Koizumi W, Narahara H, Hara T, Takagane A, Akiya T, Takagi M, Miyashita K, Nishizaki T, Kobayashi O, Takiyama W (2008). S-1 plus cisplatin versus S-1 alone for first-line treatment of advanced gastric cancer (SPIRITS trial): a phase III trial. Lancet Oncol.

[6] McMillan DC (2009). Systemic inflammation, nutritional status and survival in patients with cancer. Curr Opin Clin Nutr Metab Care.

[7] Schreiber RD, Old LJ, Smyth MJ (2011). Cancer immunoediting: integrating immunity’s roles in cancer suppression and promotion. Science.

[8] Balkwill F, Mantovani A (2001). Inflammation and cancer: back to Virchow?. Lancet.

[9] Huang XZ, Chen WJ, Zhang X, Wu CC, Zhang CY, Sun SS, Wu J (2017). An elevated platelet-to-lymphocyte ratio predicts poor prognosis and Clinicopathological characteristics in patients with colorectal Cancer: a meta-analysis. Dis Markers.

[10] Lai Q, Melandro F, Larghi Laureiro Z, Giovanardi F, Ginanni Corradini S, Ferri F, Hassan R, Rossi M, Mennini G (2018). Platelet-to-lymphocyte ratio in the setting of liver transplantation for hepatocellular cancer: a systematic review and meta-analysis. World J Gastroenterol.

[11] Li W, Tao L, Lu M, Xiu D (2018). Prognostic role of platelet to lymphocyte ratio in pancreatic cancers: a meta-analysis including 3028 patients. Medicine (Baltimore).

[12] Moher D, Liberati A, Tetzlaff J, Altman DG (2009). Preferred reporting items for systematic reviews and meta-analyses: the PRISMA statement. PLoS Med.

[13] Wells GSB, O’Connell D (2009). The Newcastle-Ottawa Scale (NOS) for assessing the quality of nonrandomised studies in meta-analyses.

[14] Ades AE, Lu G, Higgins JP (2005). The interpretation of random-effects meta-analysis in decision models. Med Decis Mak.

[15] DerSimonian R, Laird N (1986). Meta-analysis in clinical trials. Control Clin Trials.

[16] Deeks JJ, Higgins JP, Altman DG, GS HJ (2008). Analysing Data and Undertaking Meta-Analyses. Cochrane Handbook for Systematic Reviews of Interventions.

[17] Higgins JP, Thompson SG, Deeks JJ, Altman DG (2003). Measuring inconsistency in meta-analyses. Bmj.

[18] Tobias A (1999). Assessing the influence of a single study in the meta-analysis estimate. Stata Tech Bull.

[19] Deeks JJ, Altman DG, Bradburn MJ (2001). Statistical methods for examining heterogeneity and combining results from several studies in meta-analysis. Syst Rev Health Care.

[20] Egger M, Davey Smith G, Schneider M, Minder C (1997). Bias in meta-analysis detected by a simple, graphical test. Bmj.

[21] Begg CB, Mazumdar M (1994). Operating characteristics of a rank correlation test for publication bias. Biometrics.

[22] Aldemir MN, Turkeli M, Simsek M, Yildirim N, Bilen Y, Yetimoglu H, Bilici M, Tekin SB (2015). Prognostic value of baseline neutrophil-lymphocyte and platelet-lymphocyte ratios in local and advanced gastric Cancer patients. Asian Pac J Cancer Prev.

[23] Aliustaoglu M, Bilici A, Ustaalioglu BB, Konya V, Gucun M, Seker M, Gumus M (2010). The effect of peripheral blood values on prognosis of patients with locally advanced gastric cancer before treatment. Med Oncol.

[24] Deng Q, He B, Liu X, Yue J, Ying H, Pan Y, Sun H, Chen J, Wang F, Gao T (2015). Prognostic value of pre-operative inflammatory response biomarkers in gastric cancer patients and the construction of a predictive model. J Transl Med.

[25] Feng F, Sun L, Zheng G, Liu S, Liu Z, Xu G, Guo M, Lian X, Fan D, Zhang H (2017). Low lymphocyte-to-white blood cell ratio and high monocyte-to-white blood cell ratio predict poor prognosis in gastric cancer. Oncotarget.

[26] Fuentes HE, Oramas DM, Paz LH, Wang Y, Andrade XA, Tafur AJ (2018). Venous thromboembolism is an independent predictor of mortality among patients with gastric Cancer. J Gastrointest Cancer.

[27] Gong W, Zhao L, Dong Z, Dou Y, Liu Y, Ma C, Qu X (2018). After neoadjuvant chemotherapy platelet/lymphocyte ratios negatively correlate with prognosis in gastric cancer patients. J Clin Lab Anal.

[28] Gunaldi M, Goksu S, Erdem D, Gunduz S, Okuturlar Y, Tiken E, Kahraman S, Inan YO, Genc TB, Yildirim M (2015). Prognostic impact of platelet/lymphocyte and neutrophil/lymphocyte ratios in patients with gastric cancer: a multicenter study. Int J Clin Exp Med.

[29] Hsu JT, Liao CK, Le PH, Chen TH, Lin CJ, Chen JS, Chiang KC, Yeh TS (2015). Prognostic value of the preoperative neutrophil to lymphocyte ratio in Resectable gastric Cancer. Medicine (Baltimore).

[30] Jiang N, Deng JY, Liu Y, Ke B, Liu HG, Liang H (2014). The role of preoperative neutrophil-lymphocyte and platelet-lymphocyte ratio in patients after radical resection for gastric cancer. Biomarkers.

[31] Kim EY, Lee JW, Yoo HM, Park CH, Song KY (2015). The platelet-to-lymphocyte ratio versus neutrophil-to-lymphocyte ratio: which is better as a prognostic factor in gastric Cancer?. Ann Surg Oncol.

[32] Lee S, Oh SY, Kim SH, Lee JH, Kim MC, Kim KH, Kim HJ (2013). Prognostic significance of neutrophil lymphocyte ratio and platelet lymphocyte ratio in advanced gastric cancer patients treated with FOLFOX chemotherapy. BMC Cancer.

[33] Lian L, Xia YY, Zhou C, Shen XM, Li XL, Han SG, Zheng Y, Mao ZQ, Gong FR, Wu MY (2015). Application of platelet/lymphocyte and neutrophil/lymphocyte ratios in early diagnosis and prognostic prediction in patients with resectable gastric cancer. Cancer Biomark.

[34] Liu X, Sun X, Liu J, Kong P, Chen S, Zhan Y, Xu D (2015). Preoperative C-reactive protein/albumin ratio predicts prognosis of patients after curative resection for gastric Cancer. Transl Oncol.

[35] Petrillo A, Laterza MM, Tirino G, Pompella L, Ventriglia J, Pappalardo A, Famiglietti V, Martinelli E, Ciardiello F, Orditura M (2018). Systemic-inflammation-based score can predict prognosis in metastatic gastric cancer patients before first-line chemotherapy. Future Oncol.

[36] Ramos-Esquivel A, Cordero-Garcia E, Brenes-Redondo D, Alpizar-Alpizar W. The neutrophil-lymphocyte ratio is an independent prognostic factor for overall survival in Hispanic patients with gastric adenocarcinoma. J Gastrointest Cancer. 2018. PMID: 30003495. 10.1007/s12029-018-0134-z 30003495

[37] Saito H, Kono Y, Murakami Y, Shishido Y, Kuroda H, Matsunaga T, Fukumoto Y, Osaki T, Ashida K, Fujiwara Y (2018). Prognostic significance of platelet-based inflammatory indicators in patients with gastric Cancer. World J Surg.

[38] Song S, Li C, Li S, Gao H, Lan X, Xue Y (2017). Derived neutrophil to lymphocyte ratio and monocyte to lymphocyte ratio may be better biomarkers for predicting overall survival of patients with advanced gastric cancer. Onco Targets Ther.

[39] Sun KY, Xu JB, Chen SL, Yuan YJ, Wu H, Peng JJ, Chen CQ, Guo P, Hao YT, He YL (2015). Novel immunological and nutritional-based prognostic index for gastric cancer. World J Gastroenterol.

[40] Sun X, Liu X, Liu J, Chen S, Xu D, Li W, Zhan Y, Li Y, Chen Y, Zhou Z (2016). Preoperative neutrophil-to-lymphocyte ratio plus platelet-to-lymphocyte ratio in predicting survival for patients with stage I-II gastric cancer. Chin J Cancer.

[41] Tang C, Cheng X, Yu S, Wang Y, Hou J, Li Q, Shen Z, Liu T, Cui Y (2018). Platelet-to-lymphocyte ratio and lymphocyte-to-white blood cell ratio predict the efficacy of neoadjuvant chemotherapy and the prognosis of locally advanced gastric cancer patients treated with the oxaliplatin and capecitabine regimen. Onco Targets Ther.

[42] Wang F, Liu ZY, Xia YY, Zhou C, Shen XM, Li XL, Han SG, Zheng Y, Mao ZQ, Gong FR (2015). Changes in neutrophil/lymphocyte and platelet/lymphocyte ratios after chemotherapy correlate with chemotherapy response and prediction of prognosis in patients with unresectable gastric cancer. Oncol Lett.

[43] Wang Jin, Qu Jinglei, Li Zhi, Che Xiaofang, Liu Jing, Teng Yuee, Jin Bo, Zhao Mingfang, Liu Yunpeng, Qu Xiujuan (2017). Pretreatment platelet-to-lymphocyte ratio is associated with the response to first-line chemotherapy and survival in patients with metastatic gastric cancer. Journal of Clinical Laboratory Analysis.

[44] Wang K, Diao F, Ye Z, Zhang X, Zhai E, Ren H, Li T, Wu H, He Y, Cai S, Chen J (2017). Prognostic value of systemic immune-inflammation index in patients with gastric cancer. Chin J Cancer.

[45] Wang Q, Yang Y, Zhang YP, Zou Z, Qian X, Liu B, Wei J (2014). Prognostic value of carbohydrate tumor markers and inflammation-based markers in metastatic or recurrent gastric cancer. Med Oncol.

[46] Wen J, Bedford M, Begum R, Mitchell H, Hodson J, Whiting J, Griffiths E (2018). The value of inflammation based prognostic scores in patients undergoing surgical resection for oesophageal and gastric carcinoma. J Surg Oncol.

[47] Zhang LX, Wei ZJ, Xu AM, Zang JH (2018). Can the neutrophil-lymphocyte ratio and platelet-lymphocyte ratio be beneficial in predicting lymph node metastasis and promising prognostic markers of gastric cancer patients? Tumor maker retrospective study. Int J Surg.

[48] Zhang Y, Lu JJ, Du YP, Feng CX, Wang LQ, Chen MB (2018). Prognostic value of neutrophil-to-lymphocyte ratio and platelet-to-lymphocyte ratio in gastric cancer. Medicine (Baltimore).

[49] Zhou X, Xu L, Huang Z, Zhang L, Zhang H, Zhu W, Liu P (2016). The hematologic markers as prognostic factors in patients with resectable gastric cancer. Cancer Biomark.

[50] Duval S, Tweedie R (2000). A nonparametric “trim and fill” method of accounting for publication bias in meta-analysis. J Am Stat Assoc.

[51] Gu X, Gao XS, Cui M, Xie M, Peng C, Bai Y, Guo W, Han L, Gu X, Xiong W (2016). Clinicopathological and prognostic significance of platelet to lymphocyte ratio in patients with gastric cancer. Oncotarget.

[52] Zhou X, Du Y, Huang Z, Xu J, Qiu T, Wang J, Wang T, Zhu W, Liu P (2014). Prognostic value of PLR in various cancers: a meta-analysis. PLoS One.

[53] Xu Z, Xu W, Cheng H, Shen W, Ying J, Cheng F, Xu W (2016). The prognostic role of the platelet-lymphocytes ratio in gastric Cancer: a meta-analysis. PLoS One.

