# Supplementary material for: The prognostic role of platelet-to-lymphocyte ratio on overall survival in gastric cancer: a systematic review and meta-analysis
Source: BMC Gastroenterol. 2020 Jan 20;20:16. doi: 10.1186/s12876-020-1167-x (PMC6971934; doi:10.1186/s12876-020-1167-x)
Supplement: Supplementary file 1 — Additional file 1. Subgroup analyses for overall survival. Subgroup analyses for overall survival based on study design, country, sample size, percent male, mean age, treatment strategy, disease status, cutoff value and study quality. [file 12876_2020_1167_MOESM1_ESM.docx]

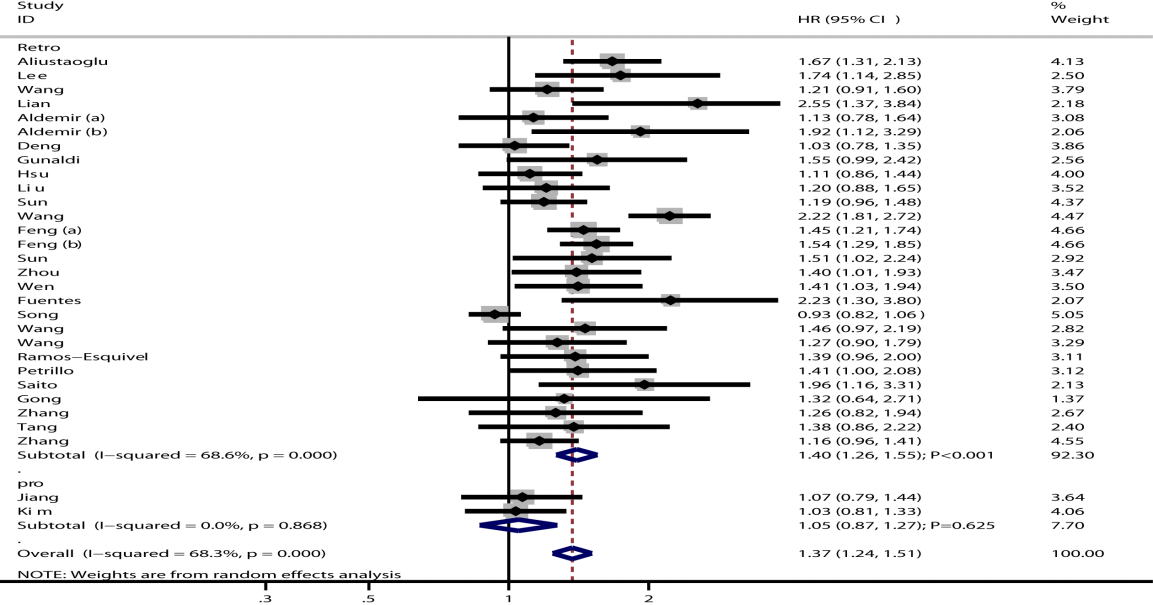


Figure S1. Subgroup analyses for overall survival based on study design


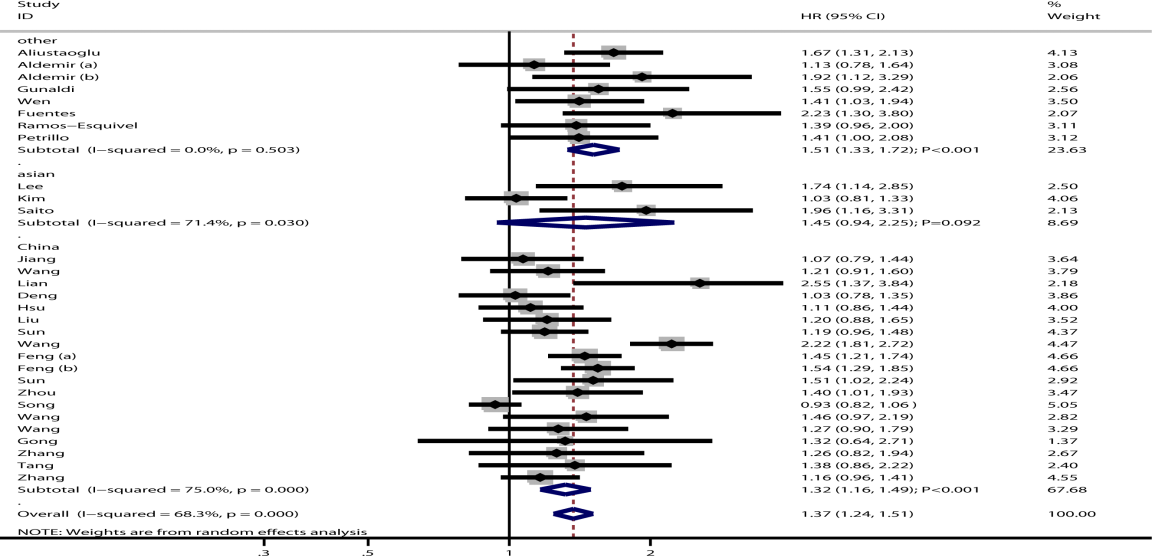


Figure S2. Subgroup analyses for overall survival based on country


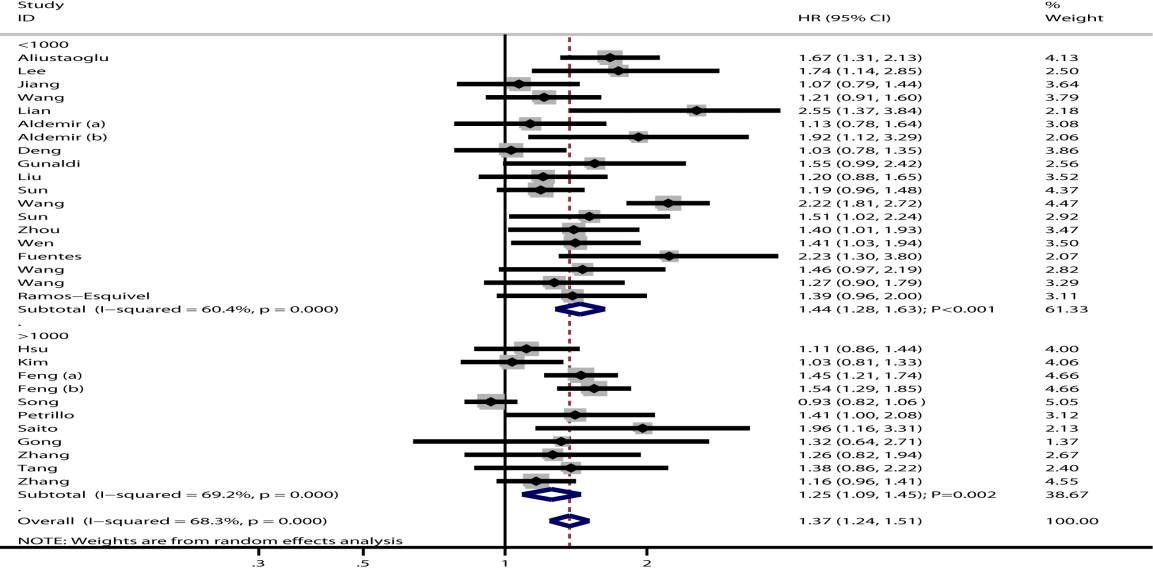


Figure S3. Subgroup analyses for overall survival based on sample size


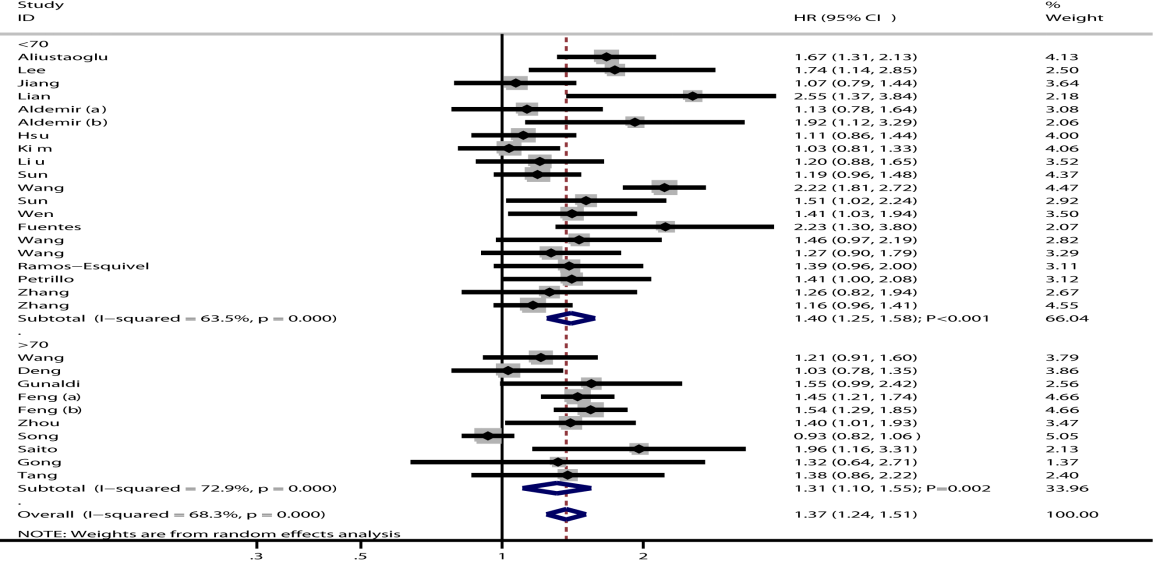


Figure S4. Subgroup analyses for overall survival based on percent male


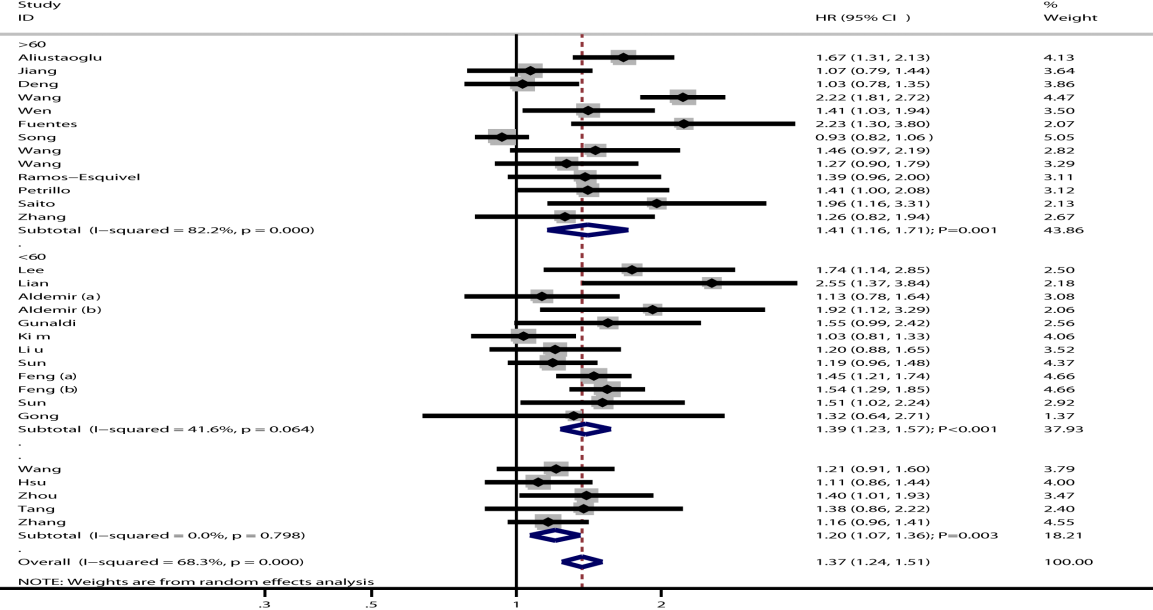


Figure S5. Subgroup analyses for overall survival based on mean age


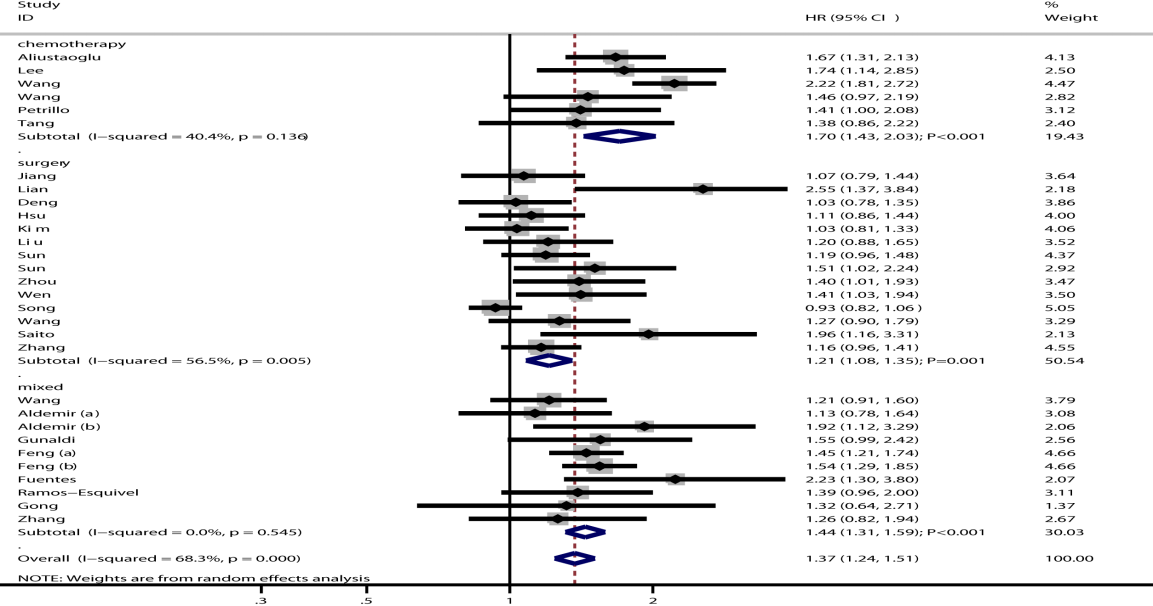


Figure S6. Subgroup analyses for overall survival based on treatment strategy


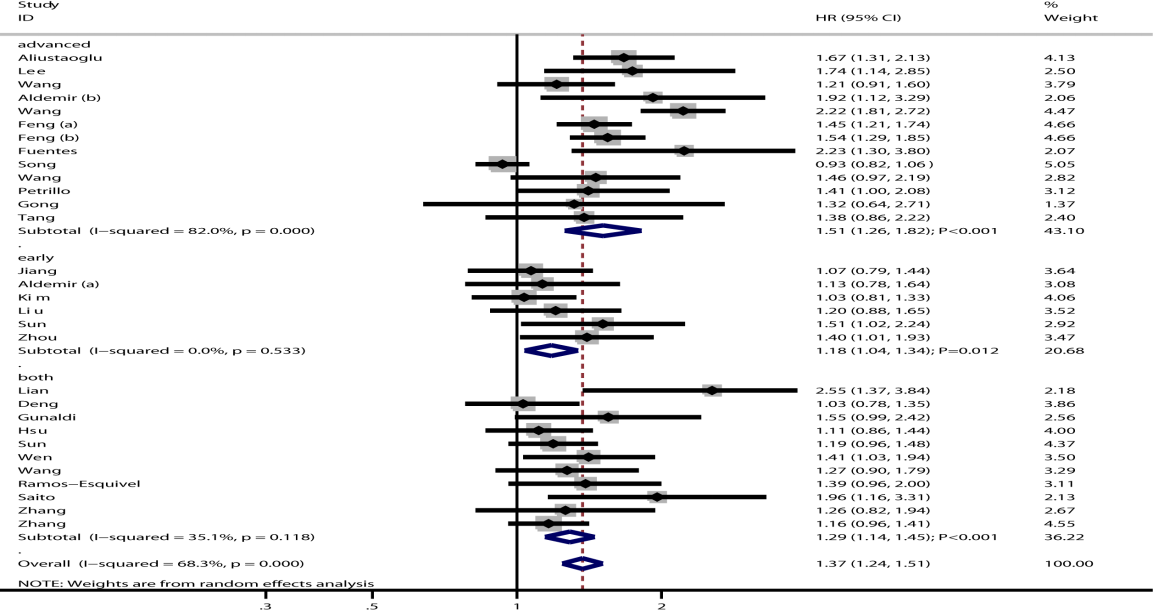


Figure S7. Subgroup analyses for overall survival based on disease status


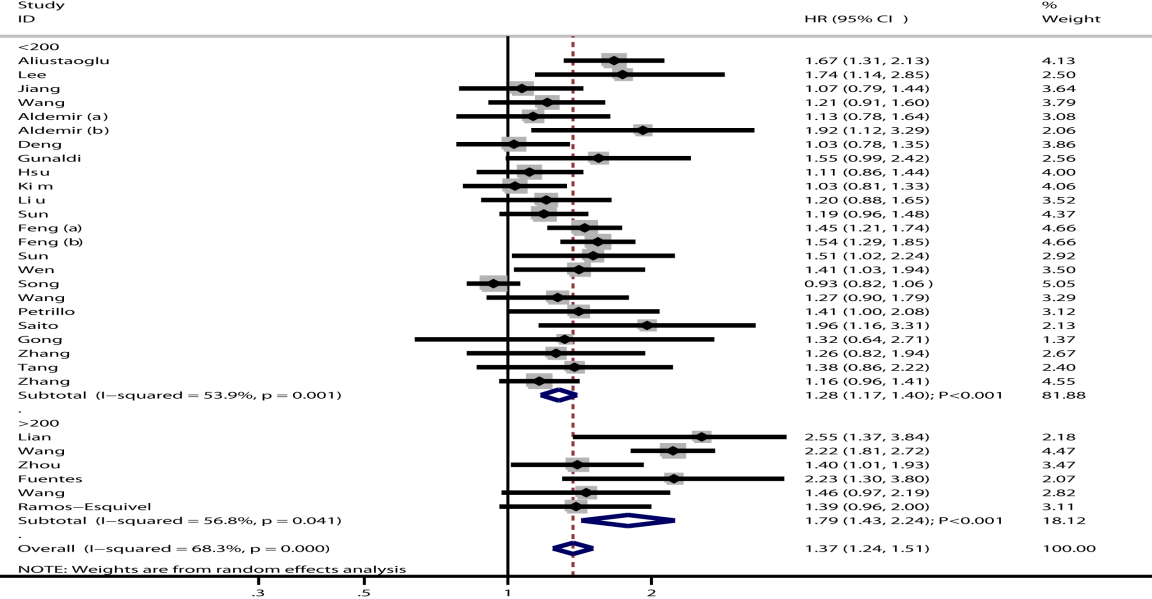


Figure S8. Subgroup analyses for overall survival based on cutoff value


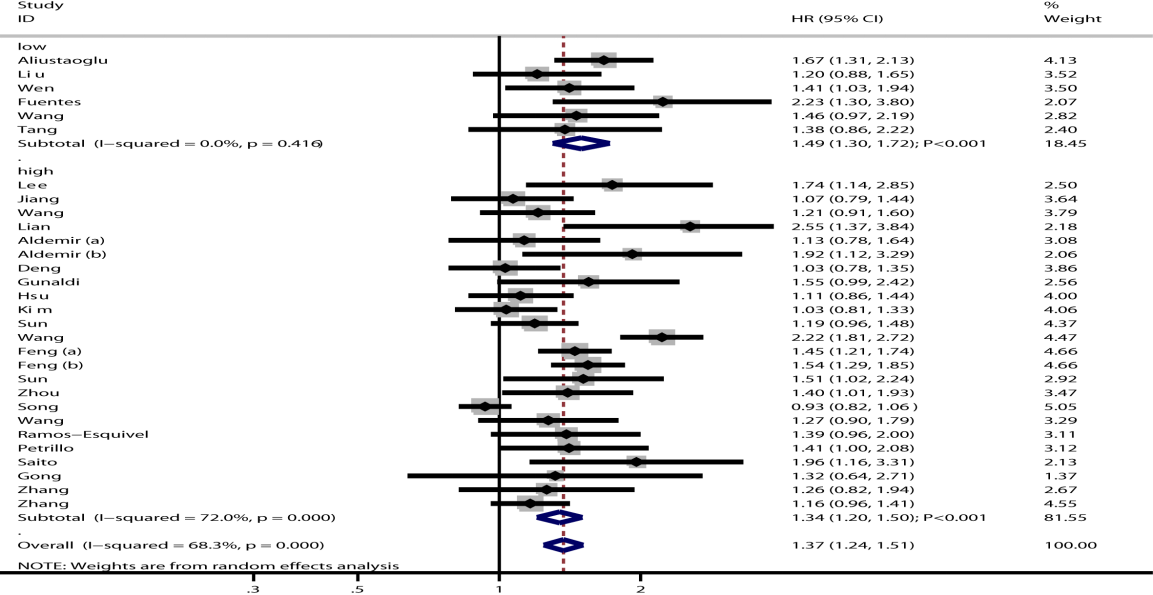


Figure S9. Subgroup analyses for overall survival based on study quality
